# Social disorganization and history of child sexual abuse against girls in sub-Saharan Africa: a multilevel analysis

**DOI:** 10.1186/1472-698X-13-33

**Published:** 2013-08-07

**Authors:** Ismail Yahaya, Olalekan A Uthman, Joaquim Soares, Gloria Macassa

**Affiliations:** 1 Department of Public Health Sciences, Mid Sweden University, Sundsvall, Sweden; 2 London School of Hygiene and Tropical Medicine, London, UK; 3 Birmingham Heart of England NHS Foundation Trust, Birmingham, UK; 4 Warwick-Centre for Applied Health Research and Delivery (WCAHRD), Division of Health Sciences, Warwick Medical School, The University of Warwick, Coventry CV4 7AL, United Kingdom; 5 Liverpool School of Tropical Medicine, International Health Group, Liverpool, Merseyside, UK; 6 Division of Social Medicine, Department of Public Health Foundation Sciences, Karolinska Institute, Stockholm, Sweden; 7 Department of Occupational and Public Health Sciences, University of Gavle, Gavle, Sweden

**Keywords:** Childhood sexual abuse, Sub-Saharan Africa, Socio-demographic factors, Demographic and health survey, Neighborhood, Social disorganization

## Abstract

**Background:**

Child sexual abuse (CSA) is a considerable public health problem. Less focus has been paid to the role of community level factors associated with CSA. The aim of this study was to examine the association between neighbourhood-level measures of social disorganization and CSA.

**Methods:**

We applied multiple multilevel logistic regression analysis on Demographic and Health Survey data for 6,351 adolescents from six countries in sub-Saharan Africa between 2006 and 2008.

**Results:**

The percentage of adolescents that had experienced CSA ranged from 1.04% to 5.84%. There was a significant variation in the odds of reporting CSA across the communities, suggesting 18% of the variation in CSA could be attributed to community level factors. Respondents currently employed were more likely to have reported CSA than those who were unemployed (odds ratio [OR] = 2.05, 95% confidence interval [CI] 1.48 to 2.83). Respondents from communities with a high family disruption rate were 57% more likely to have reported CSA (OR=1.57, 95% CI 1.14 to 2.16).

**Conclusion:**

We found that exposure to CSA was associated with high community level of family disruption, thus suggesting that neighbourhoods may indeed have significant important effects on exposure to CSA. Further studies are needed to explore pathways that connect the individual and neighbourhood levels, that is, means through which deleterious neighbourhood effects are transmitted to individuals.

## Background

Numerous studies from Africa and the rest of the world had shown that child sexual abuse (CSA) is a considerable public health problem [1]. However, until recently little attention has been paid to CSA in sub-Saharan Africa (SSA). Most of the peer-reviewed research on the sexual abuse of children in SSA is largely confined to the Republic of South Africa [2] while other reported studies in SSA are in the context of school. The current data on Africa from the World Health Organization Global School-based Student Health Survey estimated lifetime prevalence of sexual abuse among primary students aged between 13–15 years in the five countries surveyed in SSA to range from 9% to 33% [3]. In a cross study comparison of prevalence of CSA in South Africa, between 3.2% and 7.1% of all respondents report experiencing unwanted or forced sexual intercourse as a child [2]. In Swaziland, the prevalence of sexual violence before 18 years of age was 33.2% among participants aged 13–24 years [1]. While the understanding of the burden of sexual abuse in children and its relationship with adverse health behaviour has increased globally, such studies in children are nonexistent in Africa. In addition, the nature and causes of CSA is complicated due to many factors, including sexual behaviours.

When dealing with sexual behaviours, it is widely believed that focusing on individual levels ignores the broader social context within which sexual behaviour occurs. Previous studies that have investigated factors affecting CSA in SSA were based solely on the assessment of the impact of individual level variables [4-10]. However, other violence research has indicated the importance of community level factors as well as measures of social disorganization and experience of sexual violence [11]. It is important to examine whether this relationship is applicable to CSA. Thus, this study draws upon social disorganization theory [12] to examine and better understand community characteristics that may predict CSA. Although originally concerned with community conditions like delinquency and crime, social disorganization theory offers potentially important insights concerning how the characteristics of communities might be related to sexual violence. This study relies on a framework centred on the social disorganization theory (or model) to investigate the impact of neighborhood level factors on childhood sexual abuse. The framework conceptualises CSA as a multifaceted phenomenon based on interplay of individual, family, community and societal factors. In addition, the model takes into account measures of social disorganization and their role in influencing CSA.

Social disorganization identifies neighbourhood poverty, residential instability, family disruption, population density and proximity to urban areas as key structural factors that diminish community-level self-regulatory capacity [12]. The social disorganization thesis argues that communities with strong informal social networks are able to monitor and regulate sexual violent behavior [13]. Consequently, structural factors that increase the complexity of community social organization and undermine informal social networks enhance the range of sexual violent behaviours pursued by residents [14,15]. Poverty reduces the resources necessary to sustain basic institutions like the family and organizations in neighbourhoods [16]. Social disorganization theory hypothesizes that the disruptive effects of immigration, industrialisation and urbanisation lead to changes in the social structure of neighbourhoods via ethnic diversity, residential instability and neighbourhood poverty. The resulting structural changes diminish the social cohesion of neighbourhoods and reduce the power of the social norm and the informal social control required to regulate deviant behaviour. This can result in CSA. The theory proposed that high ethnic diversity gives rise to social isolation [17]. This in turn leads to structural barriers and cultural adaptations that undermine social organization. Shaw and McKay [12] also traced social disorganization to conditions endemic to the urban areas that were the only places the newly arriving poor could afford to live, resulting in a high rate of turnover in the population (residential instability). These high levels of residential turnover can disrupt existing social networks. Urbanisation has been found to be negatively associated with the coherency of the normative environment [18]. Increasing urbanisation may give rise to an environment facilitating higher levels of sexual violent activity by creating greater anonymity with minimal risk of being “found out” [18]. Non-traditional family structures, such as female-headed (matriarchal) households for example, have been linked to social disorganization. Social disorganization has received support from research conducted on extramarital sexual activity of Zambian men [13]. Research on extramarital sex is supported by Bishai and colleageus [14] who found Ugandan men in ethnically heterogeneous communities to be more likely to report such activities.

To the best of our knowledge, there has been no multilevel study performed to date that examined the association between community-level measures of social disorganization and experience of CSA in the context of SSA.

Thus, the aim of this study is to answer the following research questions:

1. Do neighbourhoods and countries differ in terms of the risks of CSA?

2. Are neighbourhood-level measures of social disorganization associated with self-reported CSA after adjustment for individual-level variables?

## Methods

The study used data from the Demographic and Health Surveys (DHS) conducted in six countries in sub-Saharan Africa (Ghana, Liberia, Nigeria, Uganda, Zambia and Zimbabwe) between 2006 and 2008. The six countries were chosen because they met the selection criteria of recent surveys during the past 10 years and the availability of data sets on sexual violence. DHS surveys were designed to collect good quality, nationally representative data on demographic and health indicators of women and members of their households. They are usually well conducted with a high response rate (average of 96%). Methods and data collection procedures have been published elsewhere [19]. Briefly, the survey utilised a two-stage cluster sampling design. The first stage involved taking up enumeration areas from census files while in the second stage, a sample of households is drawn from a current an updated list of households within each enumeration area. Every survey is stratified by urban and rural status and additionally by country-specific geographic or administrative regions. A standardised questionnaire was administered by interviewers to all female participants aged between 15 and 49 years in the selected households. To ensure standardisation and comparability across sites and time, DHS surveys employ intense interviewer training, standardised measurement tools and techniques, an identical core questionnaire and instrument pretesting [20]. The number of women included in the six DHS surveys ranged from 4,916 in Ghana to 33,885 in Nigeria. The DHS survey was implemented by respective national implementing agencies with technical assistance from ICF Macro International Inc (Calverton, MD).

### Outcome variable

To be included in the analysis, the respondents were required to meet the following two criteria: The respondents must be 18 years or younger and must be principle resident at the place where the survey interview was conducted. Furthermore, for this study, CSA was defined as sexual violence on or before the age of 18 years. To assess if participants were sexually abused in childhood, all eligible women were asked the following questions: “At any time in your life, as a child or as an adult, has anyone forced you in any way to have sexual intercourse or perform any other sexual acts?” The two possible outcomes for the questions were “yes” or “no”. Respondents who said yes were then asked questions about the age at which this first happened and the person who committed the act. Respondents who gave an affirmative reply and if the violence occurred when they were under the age of 18 years, were considered as cases of CSA and coded as “1” while those who gave a negative response or if the abuse occurred after the age of 18 years, formed the other group of the dichotomy and were coded “0”. All the women who did not respond to the question were excluded.

### Independent variables

#### Individual level factors

The following individual-level factors were included: education (no education, primary, secondary or higher); marital status (never married, currently married and formerly married) and occupation (working or not working). DHS did not collect direct information on household income and expenditure. We used DHS wealth index as a proxy indicator for socioeconomic position. The methods used in calculating DHS wealth index have been described elsewhere [21-23]. Briefly, an index of economic status for each household was constructed using principal components analysis based on the following household variables: number of rooms per house, ownership of car, motorcycle, bicycle, fridge, television and telephone as well as any kind of heating device. From these criteria the DHS wealth index quintiles (poorest, poor, middle, rich, and richest) were calculated and used in the subsequent modelling.

#### Community-level factors

(1) $$\text{Ethnic diversity index}=1-\sum_{i=1}^{n}{\left[\frac{x_{i}}{y}\right]}^{2}$$

where:

1. *Neighbourhood poverty:* percentage of households below 20% of wealth index [24].

2. *Female-headed households (family disruption)*, expressed as percentages of households headed by a female in an area.

3. *Residential mobility/instability* was defined as the proportion of households occupied by persons who had moved from another dwelling during the previous 5 years [25,26].

4. *Place of residence* was defined as either urban or rural, as administratively defined by each country.

5. *Population density (average household size)* was operationalised as the median household size in a community.

6. *Ethnic diversity* - an index of ethnic diversity was created using a formula (equation 1) that captures both the number of different groups in an area and the relative representation of each group (23):

*x*_*i *_= population of ethnic group *i* of the area,

*y* = total population of the area,

*n* = number of ethnic groups in the area

Scores can range from 0 to approximately 1. For clarity of interpretation, each diversity index is multiplied by 100; the larger the index, the greater diversity there is in the area. If an area’s entire population belongs to one ethnic group, then an area has zero diversity. An area’s diversity index increases to 100 when the population is evenly divided into ethnic groups.

Country of residence was also included as a categorical variable. The country was included as a partial control variable to control for effects of unknown factors due to potential differences across the six countries.

#### Ethics

This study was based on an analysis of existing survey data with all identifier information removed. The survey was approved by the Ethics Committee of the ICF Macro at Calverton in the USA and by the National Ethics Committees in their respective countries. All study participants gave informed consent before participation and all information was collected confidentially.

### Statistical analyses

#### Descriptive analyses

In the descriptive statistics the distribution of respondents by key variables were expressed as percentages. We used unadjusted logistic regression analyses to investigate the bivariate association between each variable and CSA.

#### Modelling approaches

We specified a 2-level multilevel model for binary outcome *y,* reporting childhood sexual abuse or not, for adolescents *i* (at level 1) living in a community *j* (at level 2) of form:

$$\pi_{\mathrm{ij}}:y_{\mathrm{ij}}\sim \mathrm{Bernoulli}\left(1,\pi_{\mathrm{ij}}\right)$$

The probability of reporting CSA was related to a set of categorical predictor *X* and a random effect for each level by a logit-link function as:

$$\mathrm{logit}\left(\pi_{\mathrm{ij}}\right)=\log\left[\pi_{\mathrm{ij}}/\left(1-\pi_{\mathrm{ij}}\right)\right]=\beta_{0}+\beta_{1}X_{\mathrm{ij}}+\beta_{2}X_{j}+\mu_{0j}$$

The linear predictor on the right-hand side of the equation consisted of a fixed part (*β*_0_ + *β*_1_*X*_*ij*_ + *β*_2_*X*_*j*_) estimating the conditional coefficients for the exposure variables and one random intercepts attributable to neighbourhoods (*μ*_0*j*_) assumed to have an independent and identical distribution and variance estimated at each level. We constructed two models. The first model, an empty or unconditional model without any exposure variables, was specified to decompose the amount of variance that existed between community levels. The second (full) model controlled for all the variables simultaneously.

#### Fixed effects (measures of association)

The results of fixed effects (measures of association) were shown as odds ratios (ORs) with their 95% confidence intervals (CIs).

#### Random effects (measures of variation)

The similarity between adolescents in the same community was measured using intra-community correlation (ICC). The ICC represents the percentage of the total variance in the probability of reporting CSA that is related to the area level, i.e. measure of clustering of odds of reporting CSA in the areas. The ICC was calculated by the linear threshold (latent variable method) according to the formula used by Snijders and Boskers Bosker [27]:

$$\mathrm{ICC}=\frac{\sigma_{\mu_{0}}^{2}}{\sigma_{\mu_{0}}^{2}+\left(\pi^{2}/3\right)}$$

where $\sigma_{\mu_{0}}^{2}$ is neighbourhood (area) variance. A high ICC in the empty model indicates high clustering CSA, in the area and thus suggesting a strong area effect on CSA. A low ICC, on the other hand, expresses the existence of a weak area influence on CSA.

#### Model fit and specifications

Regression diagnostics were used to judge the goodness-of-fit of the model. They included the tolerance test for multicollinearity, its reciprocal variance inflation factors (VIF) [28,29], presence of outliers and estimates of adjusted R square of the regression model. The largest VIF greater than 10 or the mean VIF greater than 6, represent severe multicollinearity [30]. Regression estimates were calculated by means of the reweighted iterative generalised least square algorithm using MLwiN 2.20 [31]. In the multilevel logistic regression models, second order penalized quazi-likelihood estimation was used [32]. The statistical significance of covariates was calculated using the Wald test [31]. All significance tests were two-tailed and statistical significance was defined at the 5% alpha level.

## Results

### Sample characteristics

**Figure 1:**
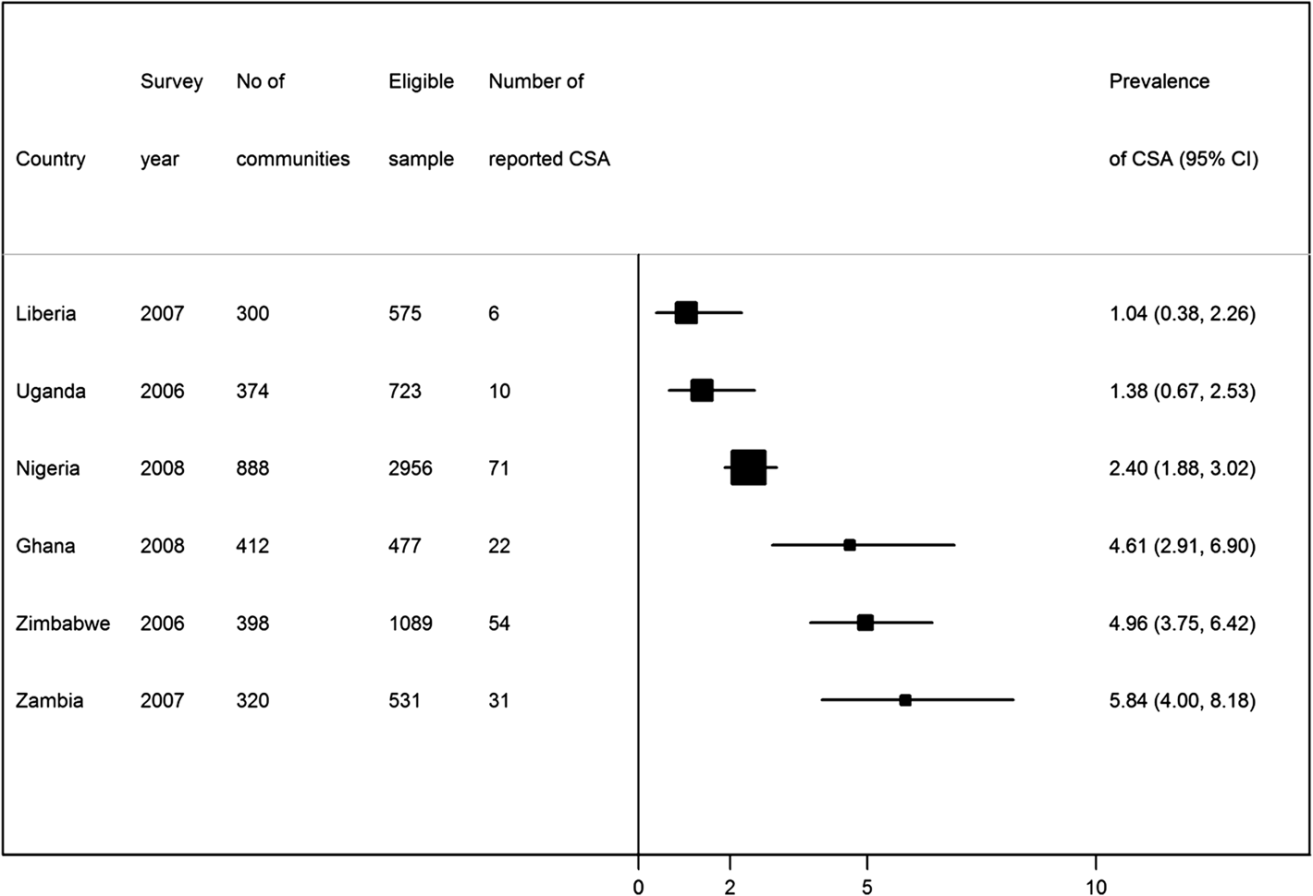
Description of demographic and health surveys data 2006–2008 in Sub-Saharan Africa by country, survey year, sample size, eligible sample and reported childhood sexual abuse (CSA).

The countries, survey year and eligible samples are shown in Figure 1. The surveys were conducted between 2006 and 2008. The number of adolescents (18 years or younger) included in the study who were permanent residence of the place at the time of the survey ranged from 477 in Ghana and 2,956 in Nigeria. The number of communities sampled ranged from as few as 300 in Liberia to as many as 888 in Nigeria. The percentage of adolescents that had experienced CSA ranged from 1.04% in Liberia to 5.84% in Zambia. Table 1 shows the characteristics of the covariates and association with CSA. Almost half (47%) of the respondents included in the final pooled sample were from Nigeria. Most of the adolescents were not married (88%); had secondary or higher education (56%), and not working (68%). There was significant association between CSA, marital status, occupation and education, but not with wealth status. The test of overall differences in prevalence of reported CSA among the six countries showed that the differential in reported CSA across the countries was statistically significant (chi-squared test [degree of freedom 5] =45.2, p=0.0001).

**Table 1:** Summary statistics and unadjusted odds ratios of the association between CSA and socioeconomic factors

	**Sample**	**Bivariate association**	
**Measures of association**	Number of adolescent (percentage)^*^	**OR (95% CI)**	**p-value**
Country			
Liberia	575 (9.1)	0.43 (0.19, 0.99)	0.047
Uganda	723 (11.4)	0.57 (0.29, 1.11)	0.099
Nigeria	2956 (46.5)	1 (reference)	
Ghana	477 (7.5)	1.96 (1.21, 3.20)	0.007
Zimbabwe	1089 (17.2)	2.52 (1.63, 3.88)	0.001
Zambia	531 (8.4)	2.12 (1.48, 3.04)	0.001
Marital status			
Never married	5566 (87.7)	1 (reference)	
Currently married	729 (11.8)	1.49 (1.00, 2.21)	0.051
Formerly married	55 (0.9)	5.90 (2.74, 12.70)	0.001
Education			
No education	763 (12.0)	1 (reference)	
Primary	2053 (32.3)	3.28 (1.57, 6.86)	0.002
Secondary or higher	3533 (55.7)	3.23 (1.57, 6.64)	0.001
Occupation			
Not working	4068 (68.2)	1 (reference)	
Working	1900 (31.8)	1.59 (1.19, 2.13)	0.002
Wealth status			
Poorest	1257 (19.8)	1 (reference)	
Poorer	1313 (20.7)	0.78 (0.52, 1.18)	0.245
Middle	1410 (22.2)	0.67 (0.44, 1.03)	0.067
Richer	1315 (20.7)	0.72 (0.47, 1.10)	0.134
Richest	1056 (16.6)	0.52 (0.32, 0.87)	0.012

^*^Percentages may not add up to 100% because of missing values.

### Measures of variability (random intercept models)

The result of the random-intercept model is shown in Table 2. The empty model (null model) shows that there was a significant variation in the odds of reporting CSA across the communities (area variance [ $\sigma_{\mu_{0}}^{2}$] =0.74, p=0.017). The intra-community correlation coefficient as implied by the intercept component variance, specified that 18% of the variation in CSA could be attributed to the community level factors. After adjusting for all the variables in the full model (Model 2), more than half (54%) of the variance in the odds of reporting CSA across communities was explained by all the variables included. The variations across communities became not statistically significant after controlling for other variables in the full model.

**Table 2:** Fixed- and random-intercept parts of multilevel logistic regression of childhood sexual abuse

	**Empty model**^**a**^	**Full model**^**b**^	
**Measures of association**			
**Individual-level**		**OR (95% CI)**	**p-value**
Country			
Liberia		0.45 (0.16, 1.33)	0.149
Uganda		0.44 (0.21, 0.90)	0.025
Nigeria		1 (reference)	
Ghana		1.62 (0.96, 2.74)	0.068
Zimbabwe		1.71 (1.14, 2.57)	0.009
Zambia		2.48 (1.53, 4.02)	0.001
Marital Status			
Never married		1 (reference)	
Currently married		2.03 (1.29, 3.19)	0.002
Formerly married		5.97 (2.55, 13.94)	0.001
Education			
No education		0.16 (0.07, 0.39)	0.001
Primary		0.84 (0.58, 1.21)	0.349
Secondary or higher		1 (reference)	
Occupation			
Not working		1 (reference)	
Working		2.05 (1.48, 2.83)	0.001
Wealth status			
Poorest		1.50 (0.70, 3.23)	0.297
Poorer		1.00 (0.51, 1.96)	0.998
Middle		0.82 (0.44, 1.55)	0.548
Richer		1.07 (0.61, 1.89)	0.807
Richest		1 (reference)	
**Community-level**			
Average household size		0.94 (0.88, 1.01)	0.091
Ethnic diversity		0.90 (0.57, 1.40)	0.629
Poverty rate		0.96 (0.61, 1.51)	0.861
Family disruption		1.57 (1.14, 2.16)	0.006
Urban (versus rural)		0.82 (0.51, 1.32)	0.413
Residential instability		1 (reference)	
**Measures of variation**			
**Community-level**			
Variance (SE)	0.74 (0.31)	0.33 (0.27)	
ICC (%)	18.3	9.1	
Explained variation (%)	Reference	54.1	

^a^Empty model – no explanatory variables.

^b^Full model 2 – Adjusted for control-, individual- and community-level factors.

*Abbreviations*: *OR* odds ratio, *CI* confidence intervals, *SE* standard error, *ICC* intra-community correlation.

### Measures of associations (fixed effects)

The results of fitting the model including individual- and community-level variables, are also displayed in Table 2. The odds of reporting CSA decreased with illiteracy. Respondents with no education were 84% less likely to have reported CSA than those with a formal education (OR = 0.16, 95% CI 0.07 to 0.39). Respondents currently employed were more likely to have reported CSA than those unemployed (OR=2.05, 95% CI 1.48 to 2.83). Compared with adolescents from Nigeria, adolescents from Uganda (OR = 0.44, 95% CI 0.21 to 0.90) were significantly less likely to have reported CSA and adolescents from Zimbabwe (OR = 1.71, 95% CI 1.14 to 2.57) and Zambia (OR = 2.48, 95% CI 1.53 to 4.02) were significantly more likely to have reported CSA. There was no evidence of differential in the odds of reporting CSA among respondents from Nigeria, Liberia and Ghana in the adjusted model. However, the test of overall differences in prevalence of reported CSA among the six countries showed that the differential in reported CSA across the countries is statistically significant (chi-squared test [degree of freedom 5] =31.54, p=0.0001). Only one community-level factor was statistically significantly associated with CSA. Respondents from communities with a high family disruption rate were 57% more likely to have reported CSA (OR=1.57, 95% CI 1.14 to 2.16).

## Discussion

To the best our knowledge, this is the first study that examined the association between both individual-level and community-level social disorganization and CSA in sub-Saharan Africa. Our results suggest that the level of family disruption in the community is associated with exposure to CSA. The associations between CSA and other measures of social disorganization were not statistically significant after adjusting for individual level factors.

Our findings are consistent with previous research carried out elsewhere, outside Africa, which had examined the association between CSA and family disruption [33-36]. There is a growing body of literature that suggests that children who experience multiple transitions in family structure may experience worse development and health outcomes compared to children raised in stable two-parent families and perhaps even worse than children raised in stable, single-parent families [34].

Results of this study suggests that family instability, especially family disruption (as a measure of community social disorganization) affects children as much as (or even more than) changes in family structure [34]. Unlike most previous studies that examined factors associated with CSA, we found evidence of significant geographical clustering in exposure to CSA. Respondents from the same area may be more similar to each other in relation to their exposure to CSA than to people from other areas [37]. Respondents living in the same neighbourhood tend to have similar exposure to CSA, which may be in part because people in the same neighbourhood are prone to common contextual influences.

This study has some caveats: Firstly, the cross-sectional nature of the data limits our ability to draw causal inferences. Secondly, the communities used in the analyses were administrative boundaries, which may not adequately capture the social context important for individual exposure to CSA. However, due to high community-level variance observed, the communities used seem to be appropriate to capture social context. DHS collects sexual violence data from females only. It would have been better if data for male victims were also available to enable comparisons between the two groups. Finally, we used self-reported measures, though the reliability and validity of this instrument is yet to be established. Despite these limitations, the strengths of the study are significant. It is a large, population-based study with national coverage from six countries with high response rates. The DHS have some important advantages when compared with other surveys. They are often nationally representative, allowing for conclusions that cover the entire nation. In addition, the same variable is operationalised in the same way and thereby making it possible for numerical values to be comparable across countries.

## Conclusion

This study found that exposure to CSA is associated with high community level of family disruption, suggesting that neighbourhoods may indeed have important effects on exposure to CSA. Further studies are needed to investigate the pathways through which neighbourhood factors interact with individual factors to influence CSA. A better understanding of the mechanisms involved might be important for designing public health interventions aimed at reducing CSA in Sub-Saharan Africa.

## Abbreviations

CSA: Childhood sexual abuse; CI: Confidence intervals; DHS: Demographic and health surveys; ICC: Intra-community correlation; OR: Odds ratio; SSA: Sub-Saharan Africa; VIF: Variance inflation factor.

## Competing interests

The authors declare that they have no competing interests.

## Authors’ contributions

IY and GM were involved in the conception of the study. IY and OU set up the statistical analysis under the supervision of GM and JS. IY was involved in the drafting of the manuscripts with contributions from all the authors. All authors revised for content and style, and all have read and approved the final manuscript.

## Pre-publication history

The pre-publication history for this paper can be accessed here:

http://www.biomedcentral.com/1472-698X/13/33/prepub
